# A student-led curriculum framework for homeless and vulnerably housed populations

**DOI:** 10.1186/s12909-020-02143-z

**Published:** 2020-07-21

**Authors:** Syeda Shanza Hashmi, Ammar Saad, Caroline Leps, Jamie Gillies-Podgorecki, Brandon Feeney, Courtney Hardy, Nicole Falzone, Doug Archibald, Tuan Hoang, Andrew Bond, Jean Wang, Qasem Alkhateeb, Danielle Penney, Amanda DiFalco, Kevin Pottie

**Affiliations:** MD MClSc CCFP FCFP; Department of Family Medicine, Bruyere Research Institute, University of Ottawa, Family Medicine Centre 75 Bruyere St, Ottawa, ON K1N 5C8 Canada

**Keywords:** Curricular framework, CanMeds, Homeless and vulnerably housed populations, Social accountability, Health equity

## Abstract

**Background:**

Medical student demands for competency based homeless health education is increasing. Indeed, humans living homeless is a treatable health and social emergency. This innovation report outlines the initial development of an education framework for homeless health.

**Methods:**

A medical student task force and educators conducted a mixed method study, including a scoping review of homeless health curriculum and competencies, a cross-country survey of medical students, and unique clinical guidelines. The task force collaborated with persons with lived experience and clinical guideline developers from the Homeless Health Research Network. The students presented at the Toronto Homeless Health Summit and refined the framework with feedback from homeless health experts.

**Results:**

The main outcome was an evidence-based Homeless Health Curriculum Framework. It uses seven core competencies; with communication, advocacy, leadership, and upstream approaches playing the strongest roles. The framework integrated the new clinical guideline (housing, income assistance, case management and addiction). In addition, it identified approaches to support mental health care with trauma informed and patient centered care. It identified public health values, clinical objectives, and case studies. The framework aims to inform the design, delivery, service learning and evaluation for medical school curriculum.

**Conclusions:**

This student-led curriculum framework can support the design, implementation, delivery and evaluation of homeless health within the undergraduate medical curriculum. The framework can lay the foundation for new doctors, research and development; support consistency across programs; and support the creation of national learning and evaluation tools.

## Background

More than half of a million Americans and a quarter of a million Canadians experience homelessness every year (US HUD, 2018 [1];). Homeless and vulnerably housed populations face increased risk for structural violence, accidental and traumatic injuries, soft tissue infections, frostbite, diabetes, cardiovascular illnesses, and mental disorders [2]. As a result, this population suffers from higher rates of preventable all-cause mortality compared to the general public [3].

Homelessness is not a traditional medical diagnosis, but rather a grave collapse of social determinants of health. Persons with lived homelessness experience could a) have experienced homelessness in the past, b) could be currently homeless, or c) could be vulnerably housed and at risk of becoming homeless. Homelessness has begun to attract the attention of medical students who are now seeking higher quality homeless-specific learning objectives and competencies. There is a lack of published evidence-based curricula that address the knowledge, skills, and attitudes required to deliver tailored and effective healthcare for homeless populations. At many medical schools, medical students rely solely on student-led outreach and advocacy initiatives. The Canadian Federation of Medical Students CFMS, for example, established a task force to develop educational initiatives around homeless health. The purpose of this innovation paper is to introduce a new framework to guide the development of homeless health undergraduate medical curriculums.

## Methods

Our initiative is built upon collaborative work from the Homeless Health Research Network and the Canadian Federation of Medical Students’ task force on homelessness. Our clinical guidelines were developed with a network of investigators, clinicians, public health experts, students and persons with lived experience of homelessness. The clinical guidelines were guided by a Delphi consensus and [1] systematic reviews on latest research evidence of interventions for persons with lived experience and they serve to outline the initial steps to improve the health of homeless populations [4]. Moreover, student leaders from the task force conducted a mixed-method curriculum quality improvement initiative that included a scoping review of the literature around homeless health curricula as well as an internal electronic survey of undergraduate medical students across Canada on the subject of homelessness and how it is currently being taught in undergraduate medicine.

Our objective was to combine student leadership and guideline development initiatives to synthesize strategies focusing on improving undergraduate medical education on homeless health. We adapted the approach used to develop medical education competencies for global health [5]. Specifically, we tailored the Canadian Medical Education Directives for Specialists (CanMEDS) competencies to reflect the clinical guidelines and evidence-based findings of the CFMS task force. As well, we sought team consensus on the values and principles underlying the framework development process.

**Fig. 1:**
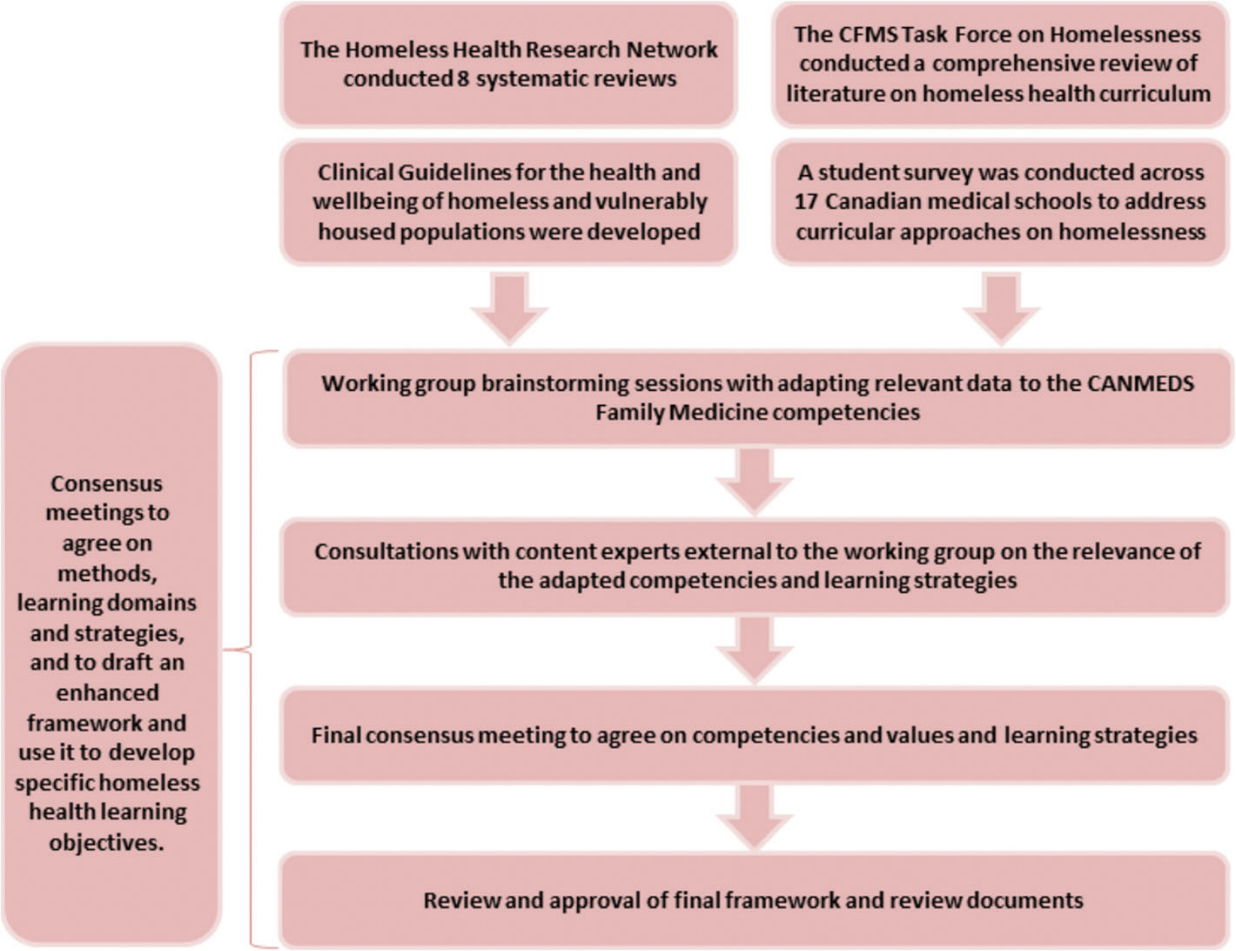
Framework for Development Approach. This describes parallel processes between the Homeless Health Research Network and the Task Force on Homelessness

Our curriculum team was composed of members of the Canadian Federation of Medical Students’ Task Force on Homelessness, content and education experts, post-graduate medical students, primary healthcare practitioners, public health experts, and persons with lived experience of homelessness from the Homeless Health Research Network. The task force used a nominal group consensus technique to devise the objectives and methodology behind our initiative (See Fig. 1 for details). The findings were presented at the Homeless Health Summit (Toronto, 2018) and received feedback from homeless health experts to further refine the framework. We engaged stakeholders with lived experience of homelessness to validate and approve our competencies, values and principles. Moreover, the consensus process allowed us to develop case studies highlighting learning approaches for homeless health training. (see Additional file 1).

## Results

The new evidence-based guidelines, quality improvement initiative, and feedback received from content, education and field experts informed the competency development and learning approaches.

### The CFMS quality improvement initiative

**Fig. 2:**
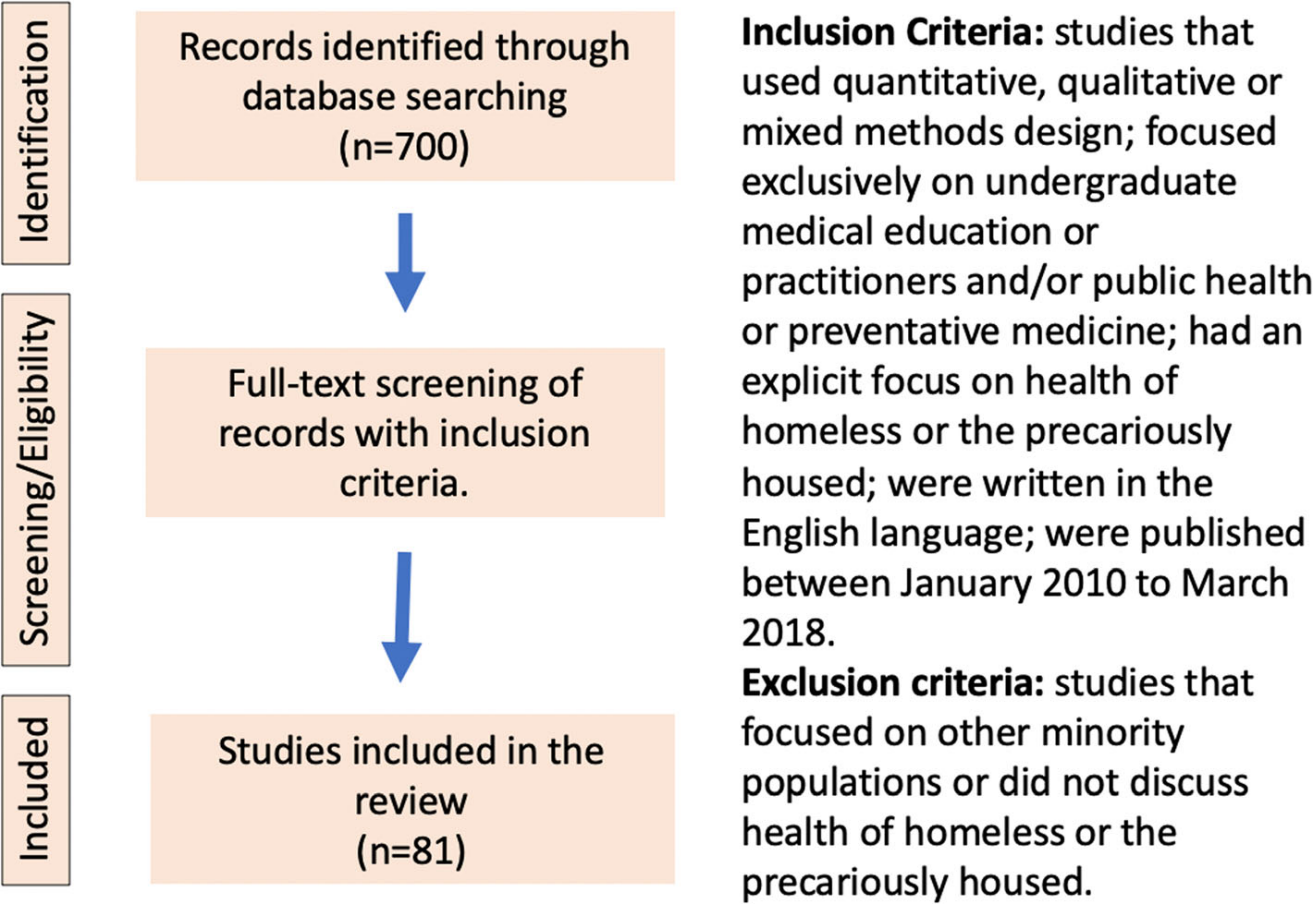
Visual representation of scoping review results

This scoping review was conducted internally within the CFMS Task Force shared with the Homeless Health Research Network for the purpose of this collaboration. Eighty-one articles included in our analysis (Fig. 2). We included studies that used quantitative, qualitative or mixed methods design; focused exclusively on undergraduate medical education or practitioners and/or public health or preventative medicine; had an explicit focus on health of homeless or the precariously housed; were written in the English language; were published between January 2010 to March 2018. We excluded studies that focused on other minority populations or did not discuss health of the homeless or the precariously housed. Findings were organized into three major themes: medical education (i.e. competencies), health professions (i.e. recent evidence-based guidelines) and policy within Canada. Findings suggest that health professionals receive little training in working with homeless populations and this may lead to negative and harmful attitudes and inappropriate care [6]. Conversely, showing empathy, cultural sensitivity, and upholding a person’s dignity builds mutual trust and improves satisfaction with care, and encourages long-term follow-up [7]. While evidence was mixed around how to integrate homeless health into medical education, evidence from the US suggested that having clinical encounters with individuals with lived homelessness experience (i.e. student-run free clinics, shelter visits, etc.) improves student attitudes, interests and preparedness [8].

**Fig. 3:**
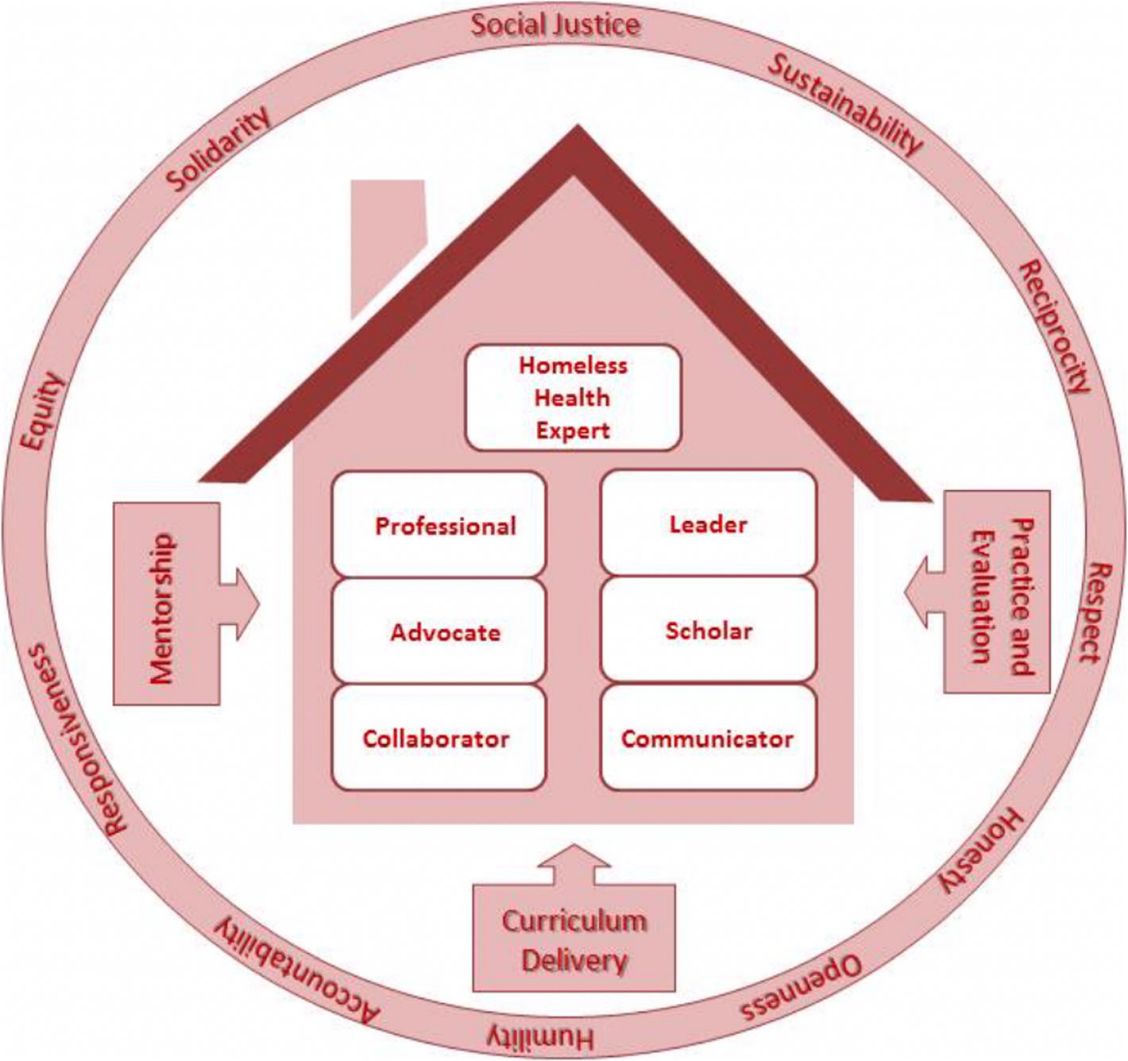
Visual representation of the homeless health education framework for undergraduate medical students

Findings of the internal survey among undergraduate medical student leaders were published internally. The survey was sent out to the 17 Canadian medical schools from September to October 2018, asking medical students with at least 1 year of medical school training to complete six open-response questions regarding their experience with education of homelessness in their medical curriculum (detailed survey in Additional file 1). Overall, 116 survey responses were received from 11 out of 17 medical institutions. In summary, respondents felt that homeless health was not being adequately taught in their medical school education and was only being addressed through self-initiated electives or extra-curricular activities. In particular, 20% of students felt that homelessness was not addressed at all in their medical curriculum, while 25% of students felt that it was minimally taught, and 9% found that it was only being addressed through self-initiated electives or extracurricular activities. On question regarding how homelessness education being delivered in medical schools, the main categories were lecture (23%), through Social Determinant of Health or population health course component (47%), electives or extra-curricular experiences (15%), case-based learning (10%), and through direct work with homeless populations (7%). Especially, 16% of responses showed that homelessness was not taught in their curriculum. When asked about medical students’ preferences around curricula addressing homeless health, responses included community outreach and service-based learning opportunities, lectures, knowledge around local resources for vulnerable populations, and elective opportunities. Particularly, suggestions in improving homelessness curriculum were categorized into pre-clerkship and clerkship stages. In the pre-clerkship stage, students wished to see more lectures on homelessness by either increasing materials and times spent on the topic (13%). Interestingly, suggestions were made to improve the education on intersections of homelessness with LGBTQ+, hidden homelessness, challenges of health service delivery to these populations, and specific population-related health needs. 43% of students suggested that they would like to see other formats of curriculum introduced, such as group sessions, community service opportunities, skills sessions, online modules, or speaker series with professionals and community outreach groups. In the clerkship stage, students expressed their wishes to have opportunities for community outreach and working directly with vulnerable populations (29%). Suggestions included mandatory sub-rotations in inner city health clinics during family medicine rotation and working in community clinics that specialize in caring for vulnerable populations in their core rotations. 20% of respondents wanted to see dedicated teaching time devoted to homelessness and appropriate clinical interventions on primary care rotations (eg. Family Medicine, CTU, Psychiatry, Emergency Medicine).

### Homeless health clinical guidelines

Our clinical guideline development process was guided by the GRADE framework and involved clinicians, investigators, public health experts, students and persons with lived experience of homelessness. Based upon expert consultations, a Delphi-consensus, and several systematic reviews, the clinical guidelines produced 5 recommendations to guide clinicians, public health professionals, and allied health practitioners in addressing homelessness as a root cause for many physical and mental health issues. The recommendations highlight the need to identify housing and income instability, complex mental health conditions, and active opioid use disorders. Similar to the ABCs of emergency medicine, homeless patients are in need of prompt linkage to evidence-based interventions such as permanent supportive housing, income assistance, intensive case management, supervised consumption facilities, and opioid agonist therapies [4].

### Identifying core values and principles

We have adapted the core values and principles that underlie family medicine education and global health curriculum framework [5]. The core team met several times, in person and via teleconferencing, to reach a consensus on these values and principles. Table 1 presents nine values that we believed underlie the homeless health curriculum framework development.

**Table 1:** Values and principles underlying homeless health and family medicine education (adapted from [5])

**Social justice**	Fair and impartial access to the benefits of society including the right to health
**Sustainability**	Living and working within the limits of available physical, natural and social resources in ways that allow living systems to thrive in perpetuity
**Reciprocity**	Multidirectional sharing and exchange of experience and knowledge among collaborating partners
**Respect**	For the history, context, values and cultures of communities with whom we engage
**Honesty and openness**	In planning and implementation of all collaborations
**Humility**	In recognizing our own values, biases, limitations and abilities
**Responsiveness and accountability**	To students and faculty and diverse communities with whom we are involved
**Equity**	Promoting the equitable distribution of resources and access, especially with respect to marginalized and vulnerable groups
**Solidarity**	Ensuring that objectives are aligned with those of the communities with which we are working

### Core competencies of homeless health

Adapting the CANMeds roles to our guidelines and student-led quality improvement findings yielded seven core competencies in the field of homeless health (Please see Additional file 1 for details). In summary, a Homeless health expert would ensure the delivery of high-quality, patient-centered, compassionate care that is built on trust and cultural safety to homeless patients with the aim of promoting better health-related attitudes and behaviors. As an advocate, a practitioner would work with patients experiencing homelessness to advocate for a system-level change within and beyond the clinical environment. As a professional, a practitioner would demonstrate a commitment to clinical excellence by adhering to ethical standards and physician-led regulations. Being a leader in homeless health requires constant contributions to the improvement of healthcare in teams, organizations, and systems. A scholar would seek continuous enhancement of knowledge by working with patients experiencing homelessness and addressing the complexity of their conditions using the best available evidence. As well a scholar would seek opportunities to create and disseminate knowledge relevant to healthcare for homeless populations. A communicator develops ethical therapeutic relationships which engages patients experiencing homelessness in the development of healthcare plans that reflect patients’ understanding of well-being, health goals, healthcare needs, and values. Finally, as a collaborator, the practitioner would work effectively in a collaborative team-based model to recognize and facilitate necessary transitions in care with other colleagues in the health professions.

### Pedagogical approaches to the curriculum framework

We used expert consultations to identify potential pedagogical domains that we could use to deliver the educational elements of this framework. The four venues that we have identified through this process were; mentorship, curriculum delivery, practice, and evaluation (Fig. 3). (Please see appendix II for case studies highlighting learning approaches for homeless health training).

## Discussion

The homeless health curriculum framework provides a foundation for local curriculum for medical schools in the US and Canada. The framework incorporates core educational elements from student-led initiatives and new evidence-based guidelines and provides enabling competencies and contextual values that underlie the educational process regarding homeless health. Adapting for the US Physician Competency Reference Set and Entrustable Professional Activities will be a first step and require participation by our American colleagues.

We drafted this framework as a stepping stone to guide medical schools and student bodies across North America in the development of pedagogical structures for homeless health in their respective curricula. One pedagogical approach that we highlight in this framework is community service learning; giving medical students the opportunity to acquire hands-on experience addressing homelessness under real world conditions. We suggest educators will want to give leadership roles for medical students to design and tailor programs in concert with expert clinicians and educators. Individual medical schools may also benefit from hosting focus groups composed of medical student leaders, educators and persons with lived homelessness experience to help apply the framework locally and enhance their curriculum to support community needs. Engaging students may drive new learning and new social accountability commitments.

Mentorship may provide the ongoing relationships that build pathways for homeless health education. Having even various mentorship relationships may help bring to life the real-world context, realities of health and social collaboration and advocacy, as well as the need to set healthy professional boundaries. Such mentorship opportunities will allow students to expand their knowledge and may facilitate vibrant learning networks across the United States and Canada. Perhaps a wise initial step would be to evaluate student and educator perceptions on local collaborations in order to develop a mentorship program that aligns with their values and principles.

## Conclusions

In summary, homelessness is a growing and deadly condition. With new clinical guidelines and student leaders with a desire to lead curriculum and social accountability, it is important to address the educational gaps that currently exist within undergraduate medical education. We hope for these guidelines to fuel further advocacy in medical education.

## Supplementary information

**Additional file 1.**

## Data Availability

Data supporting results included within our manuscript comes from anonymous feedback from medical students across Canada. This feedback is available from the corresponding author on reasonable request.

## Abbreviations

ABC: Airway, breathing, circulation
CFMS: Canadian federation of medical students
ICHA: Inner city health associates
