# Supplementary material for: A student-led curriculum framework for homeless and vulnerably housed populations
Source: BMC Med Educ. 2020 Jul 21;20:232. doi: 10.1186/s12909-020-02143-z (PMC7372848; doi:10.1186/s12909-020-02143-z)
Supplement: Supplementary file 1 — Additional file 1. [file 12909_2020_2143_MOESM1_ESM.docx]

**APPENDICES**

**Appendix 1 Homeless Health Competencies**

| **Homeless Health Expert** | |
| --- | --- |
| **Key Competencies** | **Enabling Competencies** |
| 1. Practices medicine within their defined scope of professional activity | 1.1 Demonstrates a commitment to high-quality, patient-centered, compassionate care that builds trust with patients who have lived experience of homelessness |
|  | 1.2 Cares for patients with lived experience of homelessness through the spectrum of health promotion and harm reduction; disease prevention; diagnosis and treatment, including managing life-threatening illness; acute and chronic disease management; rehabilitation; and supportive care including permanent housing, income assistance, and case management |
|  | 1.3 Carries out professional duties in the face of multiple, competing demands |
|  | 1.4 Recognizes and addresses the complexity of practice among patients with lived experience of homelessness, the uncertainty of their housing status and other social barriers that they may face, and the ambiguity inherent in medical practice |
| 2. Performs a patient-centered assessment and establishes a management plan that addresses patients’ complex needs | 2.1 Identifies relevant priorities for assessment and management such as housing and income instability, mental disorders, and unmet complex needs. These priorities should be based on the patient’s perspective, context, and medical urgency |
|  | 2.2 Elicits a thorough history, performs a physical exam, identifies signs of homelessness, income instability, and other social barriers, selects appropriate interventions that best address the patients’ needs, and refers patients to the services they choose |
|  | 2.3 Establishes goals of care in collaboration with patients and their chosen support system, which reflects the patient’s values, definition of health, and their goals for health and well-being |
|  | 2.4 Establishes a care management plan, finding common ground with the patient in defining problems, conditions, and priorities for management, and recognizing the roles of the patient, other healthcare providers and the family physician in each encounter |
|  | 2.5 Makes clinical decisions informed by the best available evidence, past experience and the patient’s perspective |
|  | 2.6 Manages complex co-existing clinical and contextual issues, both acute and chronic, often in conditions of uncertainty |
| 3. Establishes plans for ongoing care, assessment, and timely consultation when appropriate | 3.1 Works collaboratively with patients, their chosen support system, other health care colleagues, public health and key stakeholders to provide comprehensive care to individuals with lived experience of homelessness, as well as homeless populations and communities |
|  | 3.2 Implements a patient-centered care plan to ensure ongoing care, follow up on referrals, monitoring treatment, and ongoing evaluation of the need for further consultation, intervention, or care coordination |
| 4. Actively facilitates continuous quality improvement for health care and patient safety, both individually and as part of a team | 4.1 Recognizes potential health care delivery risks and patient safety incidents, especially with patients experiencing precarious housing, and works proactively to prevent harm, and remediate identified concerns and structural discrimination |
|  | 4.2 Improves patient safety, addressing human and system factors as part of a commitment to quality |
| 5. Establishes an inclusive, trusting, and culturally safe practice environment | 5.1 Demonstrates humility and openness to patients’ perspectives and experiences of homelessness and assures safety and confidentiality within their practice |
|  | 5.2 Seeks to understand culturally based health beliefs and respects patients’ understanding of health and chosen treatment plans |
|  | 5.3 Explores how the patient’s previous experiences of homelessness and adverse life events impact individual clinical encounters and interactions with the health system and incorporates this understanding in their circle of care |
| 6. Contributes generalist abilities to address complex, unmet patient or community needs, and emerging health issues, demonstrating community-adaptive expertise | 6.1 Assesses and adapts their practice based on evidence-based recommendations and population-specific needs, anticipating and planning for emerging healthcare issues, homelessness related trauma or structural barriers to accessing services in the community |
|  | 6.2 Demonstrates clinical courage (rational risk taking) and comfort with uncertainty in approaching novel and/or complex patient needs such as housing or income instabilities as well as challenges specific to homeless populations |
|  | 6.3 Creates and adjusts personal learning plans and continuing education objectives towards expanding or focusing practice as necessary, in order to develop the knowledge and skills required to provide community-adaptive care and advocacy when needed |

| **Advocate** | |
| --- | --- |
| **Key Competencies** | **Enabling Competencies** |
| 1. Responds to an individual patient’s health needs by advocating with the patient within and beyond the clinical environment | 1.1 Works with patients to overcome social barriers to good health and/ or barriers to needed health and social services or resources necessary for receiving and maintaining housing stability |
|  | 1.2 Works with patients and their chosen support system to increase opportunities to obtain safe housing, income assistance, care coordination, harm reduction and substance use services |
| 2. As a resource to their community, assesses and responds to the needs of the homeless communities or populations they serve by advocating with them as active partners for system-level change in a socially accountable manner | 2.1 Improves clinical practice by applying a process of continuous quality improvement to health promotion and disease prevention; harm reduction; diagnosis and treatment, including managing life-threatening illness; acute and chronic disease management; rehabilitation; supportive care including permanent housing, income assistance and care coordination ; intrapartum care; palliation; and end-of-life care |
|  | 2.2 Assesses community needs and identifies assets in the community or population served and contributes to a process to improve health and health equity |
|  | 2.3 Identifies specific needs of disadvantaged or marginalized populations such as Indigenous Peoples, the unsheltered homeless, homeless youth, women or migrants, including reducing barriers to access or maintain fundamental services and improving access to culturally appropriate care |

| **Professional** | |
| --- | --- |
| **Key Competencies** | **Enabling Competencies** |
| 1. Demonstrates a commitment to patients through clinical excellence and high ethical standards | 1.1 Exhibits appropriate professional behaviors and relationships in all aspects of practice, demonstrating honesty, integrity, humility, commitment, compassion, respect, altruism, respect for diversity, and maintenance of confidentiality |
|  | 1.2 Demonstrates a commitment to excellence in all aspects of practice, including integration of housing support, public policy and preventative interventions, therapeutic relationships and promotion of health and access to healthcare |
|  | 1.3 Recognizes and responds to ethical issues encountered in practice |
| 2. Demonstrates a commitment to society by recognizing and responding to societal needs in health care | 2.1 Demonstrates accountability to patients with lived experience of homelessness, allied health professionals, community based organizations and society |
| 3. Demonstrates a commitment to the profession by adhering to standards and participating in physician-led regulation | 3.1 Fulfills and adheres to professional and ethical codes, standards of practice, and laws governing practice |
|  | 3.2 Recognizes and responds to unprofessional and unethical behaviors in physicians and other colleagues in the health care professions |
|  | 3.3 Participates in peer assessment and clinical standard setting |
|  | 3.4 Fosters an environment of respect and collegiality with community-based organizations, to ensure multidisciplinary care and long-term support with community resources |
|  | 3.5 Adheres to standards of professional behaviors and boundaries with allied health professionals and community based organization |
| 4. Demonstrates a commitment to reflective practice | 4.1 Demonstrates the ability to gather, interpret, and appropriately act on information about personal performance, know one’s own limits, and seek help when needed |
|  | 4.2 Demonstrates awareness of self and an understanding of how one’s attitudes, beliefs, assumptions, values, preferences, feelings, privilege, and perspective impact their practice |
|  | 4.3 Reflects on practice events, especially critical incidents, to deepen self-knowledge and recognize when something needs to change and does it |

| **Leader** | |
| --- | --- |
| **Key Competencies** | **Enabling Competencies** |
| 1. Contributes to the improvement of comprehensive, continuity-based, and patient-centered health care delivered in teams, organizations, and systems. | 1.1 Applies the science of quality improvement to enhance the care and experiences of individuals with lived experience of homelessness |
|  | 1.2 Fosters a culture of respect and safety for patients who are experiencing homelessness. Challenges and changes negative attitudes and biases in their practice and/or institution |
|  | 1.3 Uses health data and technology informatics to improve and inform the quality of patient care across all levels of the health care system. Acknowledges the limitations in using certain technologies in the care of patients experiencing homelessness; address and mitigate these gaps however possible |
|  | 1.4 Engages and empowers individuals experiencing homelessness and their chosen support system in the process of healthcare improvement. Amplifies the voices of existing advocacy efforts across all levels of the healthcare system |
| 2. Engage in the stewardship of health care resources in the context of the patient’s needs and resources. | 2.1 Allocates health care resources for optimal patient care, based on the values and priorities of the patient. Understands that the priorities and goals of care may be different for individuals experiencing homelessness, and make clinical decisions according to the values of the patient |
|  | 2.2 Combines evidence and best practices with individual patient needs to achieve cost-appropriate care. Ensures that the patient’s social context and personal resources inform decisions involved in their care. Formulates treatment plans that are appropriate given the patient’s financial circumstances, housing status, and support systems |
| 3. Demonstrate collaborative leadership in professional practice to enhance health care for individuals with live homelessness experience h | 3.1 Advances quality care and health outcomes for patients experiencing homelessness through advocacy efforts and engaging others to impact all levels of the healthcare system |
|  | 3.2 Organizes and collaborates with others, including allied health professionals, in coalitions to achieve results that enable both individual practice and systems-level transformations |
| 4. Manages career planning, finances, and health human resources in a practice. | 4.1 Establishes and balances appropriate personal, professional and practice goals, and reassesses as needed |
|  | 4.2 Plans and manages a practice that treats individuals experiencing homelessness in a professional and ethical manner |

| **Scholar** | |
| --- | --- |
| **Key Competencies** | **Enabling Competencies** |
| 1. Engages in the continuous enhancement of their professional activities and works with individuals with lived homeless experience through ongoing learning | 1.1 Develops, implements, monitors, and revises a personal learning plan to enhance professional practice, and knowledge of relevant community resources available to individuals experiencing homelessness |
|  | 1.2 Identifies opportunities for learning and improvement by regularly reflecting on and assessing their performance using various sources, including consultations with community organizations and individuals experiencing homelessness |
|  | 1.3 Engages in collaborative learning with physicians and other allied health professionals to continuously improve personal practice and contribute to collective improvements in practice |
| 2. Teaches students, residents, the public, and other health care professionals about the resources available and important factors to consider. | 2.1 Recognizes and addresses the impact of the formal, informal, and hidden curriculum on the health of persons experiencing homelessness, the care they receive, and student perspectives and biases |
|  | 2.2 Ensures patient safety and integrity of physician-patient relationship is maintained, even when learners are involved |
|  | 2.3 Plans and delivers learning opportunities for medical students to partner and interact directly with individuals experiencing homelessness |
| 3. Integrates best available evidence into practice considering context, epidemiology of disease, comorbidity, and the complexity of patients with lived homeless experience. | 3.1 Recognizes practice uncertainty and knowledge gaps in clinical and other professional encounters, and generates focused questions that have the potential to bridge the gaps in healthcare delivery and follow-up for individuals experiencing homelessness |
|  | 3.2 Identifies, selects, and navigates pre-appraised resources and clinical practice guidelines that are relevant to the care of individuals experiencing homelessness |
|  | 3.3 Accesses and applies appropriate resources at the point of care within the clinic and in the community as appropriate |
|  | 3.4 Critically evaluates the integrity, reliability, and applicability of health-related research and literature that is relevant to individuals experiencing homelessness |
|  | 3.5 Integrates evidence into decision making in practice where relevant and useful for individuals experiencing homelessness |
| 4. Contributes to the creation and dissemination of knowledge relevant to care for individuals with lived homeless experience. | 4.1 Demonstrates an understanding of the scientific principles of research and scholarly inquiry, and the role that evidence has in the provision of health care |
|  | 4.2 Identifies and applies the ethical principles of research into providing informed consent, balancing benefits and potential harms/risks, and working with individuals with lived experience of homelessness |
|  | 4.3 Poses questions amenable to scholarly inquiry, and selects appropriate research methods from across the research continuum to answer them, including working in consultation with individuals experiencing homelessness and community organizations that interact directly with them |
|  | 4.4 Summarizes and communicates the findings of relevant research and scholarly inquiry to professional and lay audiences, including patients, their chosen support system, and communities |

| **Communicator** | |
| --- | --- |
| **Key Competencies** | **Enabling Competencies** |
| 1. Develops rapport, trust, and ethical therapeutic relationships with patients and their chosen support system (e.g. family, friends, case manager, social worker, health advocate) | 1.1 Establishes positive therapeutic relationships with patients and their chosen support system that are characterized by understanding, trust, respect, honesty, and compassion |
|  | 1.2 Optimizes the physical environment for patient comfort, dignity, privacy, engagement, and safety |
|  | 1.3 Respects patient confidentiality, privacy, and autonomy |
|  | 1.4 Listens respectfully to patients and addresses their concerns |
|  | 1.5 Responds to a patient’s non-verbal behaviors to enhance communication |
|  | 1.6 Adapts communication to the unique needs and preferences of each patient and to their clinical condition and circumstances ensuring that care is inclusive, trauma-informed and culturally safe |
| 2. Elicits and synthesizes accurate and relevant information from, and the perspectives of, patients and their chosen support system | 2.1 Engages patients to gather information about their symptoms, ideas, concerns, expectations of healthcare, and the full impact of their illness experience on their lives |
|  | 2.2 Organizes the interview in a logical sequence, attending to timing and keeping the interview on task while encouraging active patient participation |
|  | 2.3 Explores the patient’s personal life context, including barriers to stable housing, using trauma-informed conversational techniques |
|  | 2.4 Seeks and synthesizes information from other sources such as the patient’s chosen support system and/or health advocate |
|  | 2.5 Engages with patient’s chosen support system during important life events to improve understanding of the patient’s experience |
| 3. Shares health care information and plans with patients and their chosen support system | 3.1 Shares information and explanations that are clear, accurate, and timely, while checking for patient and others understanding, taking into consideration diversity of life experiences, such as previous trauma, as well as language barriers, and health literacy levels |
|  | 3.2 Discloses patient safety incidents to patients and their chosen support system accurately and appropriately |
| 4. Engages patients and their chosen support system in developing plans that reflect the patient’s understanding of well-being, health goals, health care needs, and values | 4.1 Facilitates discussions with patients and others about the treatment plan in a way that is respectful, inclusive, non-judgmental, reduces harm, and is culturally safe, including using an interpreter, Indigenous liaison, or health advocate when needed |
|  | 4.2 Assists patients with identifying, accessing, and using appropriate information and communication technologies to support their care, make informed decisions and manage their health while maintaining confidentiality |
|  | 4.3 Recognizes and respects diversity, including but not limited to the impact of homelessness, poverty, previous trauma, gender, ethnicity, religion, and cultural beliefs, on joint decision making and other interactions |
|  | 4.4 Effectively addresses challenging communication issues such as motivating behavior change, delivering bad news, and addressing disagreements and emotionally charged situations |
|  | 4.5 Provides therapeutic interventions through supportive and other counselling techniques |
|  | 4.6 Helps patients clarify their values and feelings, cope with uncertainty, and sort out their options for care |
| 5. Documents and shares written and electronic information about the medical encounter to optimize clinical decision making, patient safety, confidentiality, and privacy | 5.1 Maintains timely, clear, accurate, and appropriate written or electronic records of clinical encounters |
|  | 5.2 Presents medical information to the public or media about a medical issue when requested |
|  | 5.3 Uses electronic health records to enhance shared decision making with patients |

| **Collaborator** | |
| --- | --- |
| **Key Competencies** | **Enabling Competencies** |
| 1. Works effectively with others in a collaborative team-based model | 1.1 Establishes and maintains knowledge of important resources and referrals in the community for persons with lived homeless experiences |
|  | 1.2 Defines and negotiates overlapping and shared roles and responsibilities of allied healthcare professionals and community partners to meet homeless patients’ complex needs, such as acute and chronic care, mental health and addiction care, and housing support |
|  | 1.3 Respects diversity of roles and perspectives while ensuring integrated patient-centered care |
| 2. Cultivates and maintains positive working environments through promoting understanding, managing differences, minimizing misunderstandings, and mitigating conflicts | 2.1 Demonstrates a respectful attitude toward patients experiencing homelessness, allied health professionals and community-based organizations towards developing a positive team-based approach to providing care |
|  | 2.2 Works closely and interacts directly with community partners and individuals experiencing homelessness, such as through in-person community visits, to promote understanding, empathy, mutual trust and clinical competency |
|  | 2.3 Recognizes and reflects on one’s own contributions and limitations regarding care of patients experiencing homelessness, and seeks support from knowledgeable resources and community partners when necessary |
| 3. Recognizes and facilitates necessary transitions in care with other colleagues in the health professions, including but not limited to shared care, transfer of care, and/or handover of care to enable continuity and safety | 3.1 Determines when a transition or continuation in care is required, such as involving specialists, mental health and addiction services, and other community-based partners, and facilitates the process |
|  | 3.2 Effectively negotiates and communicates (both verbally and in writing) individual and/or shared responsibilities, through care transition plans, and novel approaches to follow up, to optimize patient safety and outcomes |

**Appendix 2 Case studies highlighting learning approaches for homeless health training:**

The following case studies serve as examples to apply the homeless health framework in undergraduate medical education.

**Mentorship approaches**

The specialized generalism required to adequately meet the demands of the unique health complexity of people experiencing homelessness is most fully nurtured within a mentorship relationship. In distinction from classroom learning and clinical (s)electives, mentoring provides the opportunity to shape complex skills over sustained periods within the context of a relationship grounded in mutual commitment and progressive exposure and depth of understanding.

Mentoring approaches can focus on individual competencies (Expert, Advocate, Communicator, etc) and/or more global mentoring models that encompass numerous individual competency domains. As many of the competencies require knowledge, values and practices that derive from the social welfare, housing, immigration, legal and other sectors, it is advisable to consider mentorship experiences from many sectors. These are opportunities not only to deepen one’s competency in that sector but to cultivate the ability to discuss health matters with colleagues beyond the healthcare sector and learn to integrate the insights and skills of this sector within the realm of healthcare. Such multi- and cross-sectoral collaboration is essential to becoming a housing-capable healthcare provider.

Trainees should consider sharing their experience with others through mutual support, student groups, learning collaboratives, advocacy initiatives and connections to mentorship experiences that have been found to be valuable. Such student-student mentorships offer valuable opportunities for professional development, as well as create a supportive framework for students to know when one is ready to share experiences with and support others.

**Curriculum delivery approaches**

Students, working with supportive teachers, are important agents of change for medical curriculum. The homeless health curriculum framework and the new CMAJ homeless health clinical guidelines (CMAJ TBA) can support students and faculty in the design, delivery and evaluation of new homeless health learning tools and curriculum. These learning tools could include student-led special interest groups, integrated problem-based learning, and new community and service-learning programs that may enhance social accountability. Concretely, for example, in 2013 student leaders and supervisors developed and evaluated a refugee health e-learning program based on the competencies, values, and refugee learning objectives (Redwood Campbell BMC 2009) and global health field experiences of faculty. (Global and Refugee e-learning). This e-learning program is now being used as a springboard curriculum tool for junior medical students interested in refugee health and related community service learning in 18 universities across Canada.

**Community service-learning approaches**

Community service learning (CSL) programs have a valuable role in providing students with opportunities to apply theoretical knowledge, refine practical skills, and continually adapt learning in various contexts. The framework in this paper can guide the development of a successful service-learning initiative involving homeless populations. For instance, one opportunity to develop a CSL initiative would be a program that pairs medical students with clients or families experiencing homelessness to help them transition back into the community. This program would allow students to support the clients and connect them with relevant community resources such as income supports (ie. assisting with budgeting, application forms, and linkage to relevant resources). Many similar CSL programs address refugee health by pairing medical students with refugee families to help them integrate into the health system and community (The University of British Columbia Faculty of Medicine Global Health 2017; Canadian Collaboration for Immigrant and Refugee Health). Service-learning programs have been shown to give students the opportunity to apply their theoretical knowledge and continuously adapt their learning to various contexts (Sabo 2015). They challenge students to develop the communication and clinical skills to work with vulnerable populations and strengthen the student’s role of health advocate as they help clients navigate a complex social system. They also provide an opportunity for students to apply their knowledge on the evidence-based guidelines on working with vulnerable populations. Finally, these programs allow students to develop leadership and collaboration skills.

**Evaluation approaches**

A professor at the University of Ottawa was given responsibility to design a global health class in which undergraduate medical students would learn about emerging global issues and their impact on the Canadian healthcare system. The professor wanted to incorporate homeless health competencies into his syllabus to demystify the distorted cognitions of treating homeless patients which some medical students might possess. Students enrolled in his course were expected to visit a homeless shelter, ask residents for a rapid medical interview and document their medical history and profile. Moreover, the students were instructed to examine the impact of housing instability on the health status of this population. The professor wanted to evaluate students’ experiences and thus instructed them to use a journal and write their perceptions before and after these encounters. After delivering their assignments and journals, the professor analyzed what the students wrote and, as expected, she found that students unanimously felt scared and anxious prior to their encounters with a homeless patient. However, by implementing some enabling competencies such as using a sensitive language, showing compassion, and displaying non-judgmental communication skills, students’ reflections and cognitions on homelessness had showed positive change. They reported that all the homeless patients they had interviewed were friendly and open about their life stories, making it easier to write the medical reports. Further, students have shown a sense of compassion and understanding of the reasons behind homelessness, which was not prevalent prior to their encounters. When reporting medical conditions, the majority of students were able to conclude that housing instability had a significantly negative impact on the health status of this vulnerable population.

**Appendix 3: Student Survey**

1. What Medical school do you attend?
2. What year of medical school are you in?
3. How has homelessness been taught in your medical school?
4. Is there a curriculum for social determinants of health?
5. How would you like to see homelessness being addressed in pre-clerkship
6. How would you like to see homelessness addressed in clerkship?
7. Have you been involved in any student groups or advocacy initiatives that address homelessness and health?
8. Is there anything else you would like to say regarding homelessness teaching in medical school?
